# Increasing Active Transportation Through E-Bike Use: Pilot Study Comparing the Health Benefits, Attitudes, and Beliefs Surrounding E-Bikes and Conventional Bikes

**DOI:** 10.2196/10461

**Published:** 2018-11-29

**Authors:** Taylor H Hoj, Jacob J Bramwell, Cameron Lister, Emily Grant, Benjamin T Crookston, Cougar Hall, Joshua H West

**Affiliations:** 1 Health Behavior Outcomes Lab Department of Public Health Brigham Young University Provo, UT United States; 2 Department of Sociology Brigham Young University Provo, UT United States

**Keywords:** physical activity, bicycling, obesity, physical fitness, cardiorespiratory fitness

## Abstract

**Background:**

The emergence of electric pedal-assist bicycles (e-bikes) presents an opportunity to increase active transportation by minimizing personal barriers of engaging in physical activity.

**Objectives:**

The aim of this study was to assess the beliefs of individuals using e-bikes for active transport and report preliminary biometric measurements while using e-bikes for physical activity compared with conventional bikes.

**Methods:**

Participants used both conventional bicycles and e-bikes to compare energy expenditure while riding on the study route. Apple smart watches were used to track each participant’s heart rate, distance, speed, and time while riding both bicycles. A total of 3 survey instruments were used to estimate beliefs: one administered before riding the bicycles, a second administered after riding a conventional bike, and the final survey completed after riding an e-bike. Survey instruments were constructed using constructs from the theory of planned behavior.

**Results:**

The study sample (N=33) included adults aged between 19 and 28 years. Paired *t* test analysis revealed that participants believed a conventional bike was more likely than an e-bike to benefit their physical health (*P*=.002) and save them money (*P*=.005), while an e-bike was perceived to be more likely than a conventional bike to save them time (*P*<.001). Paired *t* test analysis revealed participants significantly agreed more with the statement that they could ride an e-bike most days (*P*=.006) compared with a conventional bike. After participants traveled approximately 10 miles on each type of bicycle, participants’ mean average heart rate while riding the e-bike was 6.21 beats per minute lower than when riding the conventional bike (*P*=.04), but both were significantly higher than resting heart rate (*P*<.001).

**Conclusions:**

This pilot study suggests that e-bikes are an active form of transportation capable of providing much of the cardiovascular health benefits obtained during conventional bike use. E-bikes may help reduce some of the obstacles to conventional bike use, such as increased transportation time, decreased convenience, and physical fatigue.

## Introduction

### Background

Physical inactivity has been identified as a contributing factor to obesity, which is currently a leading public health issue in developed nations [1-3]. Health authorities have promoted active transportation as one possible response to addressing this epidemic [4]. Active transportation includes transportation activities that are human-powered, such as biking to work. This is distinct from many intentional exercise or fitness activities in that the purpose of active transport is primarily to get from one location to another. Substantial research in the fields of transportation, health, and psychology has helped to identify a variety of factors associated with engaging in active transport behaviors, including features in the built environment [5,6], age and gender [7], and attitudes and beliefs in a culture [8]. Though active transportation may be a promising approach to addressing obesity, it is not without its barriers. Active transportation can be made difficult because of barriers such as lack of safe walking and cycling paths, long commuting distance, limited current fitness level, lack of time, and inclement weather [7]. These barriers may be divided thematically into 2 classes: personal factors (eg, too much effort to ride a bike or a desire to wear normal clothes without getting to work sweaty) and environmental factors (eg, dangerous road or traffic conditions) [9]. As these barriers limit consistent and sustainable active transportation, innovative methods of active transportation that help to reduce or even eliminate such barriers are of interest to public health professionals.

In recent years, e-bikes have emerged, presenting a potential opportunity to encourage active transportation while reducing personal barriers to active transportation [10-12]. E-bikes operate through a small electric motor that acts as a pedal-assist, only providing assistance when the rider pedals. Because of this feature, the rider can theoretically still obtain at least a portion of the physical activity benefits of conventional cycling while reducing some of the traditional personal barriers to commuting with a conventional bicycle. Commuters may not want to exert the effort required to ride a conventional bicycle, may need to travel a longer distance, or may desire to wear normal clothing without arriving to their destination sweaty. In addition, individuals may have limited time or may not have the stamina to make the trip with a conventional bicycle. In each of these cases, the added assistance of the pedal-assist electric motor in e-bikes may reduce these barriers while still providing a portion of the health benefits associated with conventional cycling [12,13].

E-bikes also have the added benefit of being environmentally friendly, as they do not produce carbon emissions or noise pollution akin to their motorized vehicle counterparts [14,15]. In addition, they are not like motorcycles or other motorized scooters in that they can generally be ridden on bike paths and in bike lanes. If adopted widely enough, e-bikes could, therefore, reduce congestion in traffic as well as car parks, as they can also be parked with traditional bicycles.

On account of the relatively recent introduction of e-bikes, the current literature surrounding e-bikes is somewhat limited. To date, the majority of e-bike studies have focused on issues concerning safety [16-26]. Some research, however, has been focused on the potential physical health benefits of e-bikes and their potential to reduce personal barriers to traditional cycling. For example, results from a Web-based survey demonstrated that those using an e-bike to ride to work report an ability to ride greater distances while perspiring less, suggesting that e-bikes may reduce some of the personal barriers of traditional cycling [27]. Being able to ride greater distances was also confirmed in another Web-based survey of e-bike users [28]. A review of e-bike literature supports the idea that e-bikes are related to beneficial physical activity but that they also may be more dangerous than a traditional bike [29].

One study also suggested that e-bikes may have an added benefit of promoting health among individuals otherwise reluctant to engage in physical activity [30,31]. Previous e-bike studies with such populations (older individuals, obese or overweight individuals, and those who may be impacted by physical injury or impairment) have largely focused only on safety [32,33], though one study has examined e-bike use among untrained, overweight individuals [13].

Heart rate and energy expenditure is likely lower with an e-bike compared with what would be observed with a conventional bicycle, and this has been confirmed in 2 small studies, with sample sizes of 18 and 12 [34,35]. Another study of 8 individuals suggests that e-bike use results in lower oxygen consumption and exercise intensity but that moderate physical activity is still achieved [10]. Similarly, a study of 10 trained and 10 untrained individuals revealed that though power output, exercise intensity, and energy expenditure were lower with assistance from the electric motor, the exercise intensity was sufficiently high to achieve the standards for moderate-intensity health-enhancing physical activity [12]. Recent studies also suggest that e-bike commuting may improve metabolic fitness such as glucose tolerance [36] and that riders experience lower levels of perceived exertion and higher levels of enjoyment [31].

Despite these findings, there is limited research in the current literature regarding the attitudes, beliefs, and perceptions of e-bikes and their potential health benefits compared with those of conventional bikes. For this reason, an assessment of the attitudes and beliefs toward their use is needed, as even if beneficial for health reasons, it remains unclear if individuals would adopt this technology or perceive it to be of limited value.

### Objective

The purpose of this pilot study was to compare e-bikes with conventional bicycles. More specifically, this study sought to answer 2 research questions: (1) what proportion of the health benefits are retained when using an e-bike as compared with using a conventional bicycle? and (2) what are the attitudes and beliefs toward e-bikes after riding one and how do those compare with attitudes and beliefs toward conventional bicycles? In particular, this study aimed to understand attitudes and beliefs regarding personal factors that may prevent active transportation events.

## Methods

### Participants

A total of 33 participants were recruited to this pilot study through announcements in undergraduate public health courses at a large private university in the state of Utah in the United States. Cycling in this area is relatively common, with the modal share for biking to work in Utah being 0.8% in 2014, making it the 11th-highest in the country [37]. Eligibility was limited to individuals between the ages of 18 and 65 years. Exclusion criteria included the inability to complete a survey in English, the inability to ride a bicycle at moderate to vigorous intensity for 10 miles (approximately 16 km), or a medical condition preventing moderate exercise.

### Procedures

The institutional review board at Brigham Young University approved this study. Individuals desiring to participate first completed an informed consent form and then received an email link to a baseline survey with items relating to demographics, physical activity level, cycling history, as well as attitudes and beliefs about biking. Participants were then assigned a day and time to meet at a bike-park stall in the university campus. At the stall, participants were provided a heart rate monitor and global positioning system (GPS) device, a bicycle helmet, and a conventional bicycle. Participants were given instructions related to bicycle safety and bike path etiquette. They were also provided with a healthy snack and water bottle to ensure they had energy and water. Participants kept the water bottle. Participants were shown a map of the regional dedicated bike path and received detailed directions for the intended 10-mile path of travel. The bike path was generally flat, and elevation change during the ride was relatively minimal. Participants were then instructed to ride the prescribed bike path route at a comfortable speed. After completing the first ride, participants were emailed a link to a second survey with items relating to their experience, attitudes, and beliefs of riding the conventional bicycle on the study route. On a second day, participants returned to the same location to ride the study route again—this time using an e-bike. Rides were separated by an average of 6 days. Participants were again provided a heart rate monitor, GPS device, bicycle helmet, basic instructions related to bicycle safety and bike path etiquette, and a refresher on bike path directions for the same study route. In addition, participants were given instructions for the safe riding and operation of an e-bike. After completing the second ride, participants were emailed another link to the survey designed to measure their experience, attitudes, and beliefs of riding the e-bike on the study route. Participants completed rides between November 2016 and June 2017, with the majority taking place in April and May.

### Instruments and Measurements

Both conventional bicycles and e-bikes were used in this study to establish a comparison between participant’s energy expenditure while riding the study route. The conventional bikes were recreational mountain bikes equipped with 21 speeds, disc braking systems, and adjustable seat heights. The e-bikes were Specialized Turbo 2016 models equipped with 9-speeds, front suspension, disc braking systems, and adjustable seat heights.

Apple brand smart watches were used to track each participant’s heart rate, distance, speed, and time while riding both the conventional bicycle and the e-bike. A comparison of participants’ heart rate was used as a proxy measure to estimate health benefits retained during e-bike use compared with conventional bike use. Specifically, estimated maximum heart rate (MHR) was calculated by subtracting the mean age of the study group from 220. The estimated MHR was then used to establish a target average heart rate range for moderate-intensity physical activity. This range was calculated based on the target heart rate recommendations from the Centers for Disease Control and Prevention for moderate-intensity physical activity [38]. The free version of Strava, a mobile app using GPS technology available via the App Store for iOS and Apple Watch platforms, was used to measure speed and distance. During the e-bike rides, 2 participants experienced technical difficulties with the e-bikes in which the batteries were not functioning properly and, therefore, not providing assistance for the full duration of the ride. Because of this, the time, speed, distance, and heart rate measurements for these 2 participants were excluded from analysis. In addition, the heart rate measurement function of the Apple watches did not work properly for 2 participants, and their heart rate measurements were therefore excluded from analysis.

A total of 3 survey instruments, developed using the Web-based survey software provided by Qualtrics, were used in this study. Survey 1—administered before riding either of the bicycles—was used to gather basic demographic information (eg, age, ethnicity, education, income), typical personal transportation methods (eg, bus or train, car, bicycle), cycling history and experience data (eg, whether or not the participant owns a bicycle or e-bike), and information about general attitudes and beliefs regarding bicycles (eg, obstacles to riding a bicycle, social stigma). The information about attitudes and beliefs gathered in survey 1 was used to inform the development of questions in survey 2 and survey 3. Survey 2—administered after completing the ride on the conventional bike—assessed agreement with prosocial benefits of bicycle use using a 5-point Likert scale (eg, health, environment, saving time or money), social support for using cycling as a method of transportation (eg, feeling embarrassed to use a bicycle for transportation purposes), and the likelihood of using a bike under adverse conditions (eg, cold, rain, darkness, fatigue, hilly terrain, and so on). Survey 3—administered after completing the ride on the e-bike—was used to collect the same information as survey 2, but all the items reflected the participants’ experience, attitudes, and beliefs related to riding an e-bike. The questions in survey 2 and survey 3 were identical except for the fact that survey 2 asked the questions in relation to conventional bicycles, while survey 3 asked the questions in relation to e-bikes. The surveys were also subjected to standard face and content validity assessments.

The theory of planned behavior (TPB) was used as a basis for the development of the surveys [39,40]. Within the TPB, a subjective norm is an individual’s perception of social normative pressures or relevant others’ beliefs that an individual should or should not perform a particular behavior. In the case of biking, subjective norms could include perceived pressures to ride or not ride a bicycle (or e-bike). Attitudes reflect an individual’s perception or belief regarding the extent to which, for example, riding a bike will be a benefit (behavioral belief) and, secondarily, the extent to which the individual desires the outcome (outcome evaluation). Finally, the perceived behavioral control construct represents an individual’s assessment of his or her own ability to ride a bike (or e-bike) in the context of potential external barriers (eg, bad weather). These constructs provided a framework for the development of all study surveys.

### Analysis

All statistical analyses were performed using SAS version 9.4 (SAS Institute Inc). Descriptive statistics were used to summarize demographic data from survey 1. Paired *t* test statistics were calculated to compare beliefs of conventional bicycles and e-bikes as well as to compare mean heart rate and speed between conventional bicycle and e-bike use. Heart rates from each ride were also compared against the resting heart rate. A separate set of paired *t* test statistics of heart rate data stratified by gender was also conducted.

## Results

### Demographics

The majority of the participants were aged between 20 and 24 years, with the average age being 22 years. Most identified themselves as non-Hispanic whites. Most participants had completed college, but had not graduated, and approximately three-quarters of the study sample reported an annual income of less than US $30,000. Complete demographic information can be found in Table 1.

**Table 1:** Demographics of participants (N=33).

Demographics		n (%)
**Age (years)**		
	18-19	3 (9)
	20-24	27 (82)
	25-34	3 (9)
**Race**		
	Asian	2 (6)
	White	31 (94)
**Ethnicity**		
	Not Hispanic or Latino	33 (100)
**Gender**		
	Male	20 (61)
	Female	13 (36)
**Education level**		
	High school or GED^a^	1 (3)
	Some college (not graduated)	27 (82)
	2-year college degree	2 (6)
	4-year college degree	3 (9)
**Annual household income^b^**		
	Less than 30,000	24 (73)
	40,000-49,999	1 (3)
	60,000-69,999	1 (3)
	70,000-79,999	1 (3)
	100,000 or more	6 (18)

^a^GED: General Educational Development.

^b^All values are in 2017 US $.

**Table 2:** Transportation methods (N=33).

Transportation methods			n (%)
**Participants own the following**			
	Bike		20 (61)
	E-bike		0 (0)
	Car or truck		24 (73)
	Motorcycle or motor scooter		1 (3)
**What is your most frequent method of transportation?**			
	Walk		12 (36)
	Bicycle		5 (15)
	Drive		15 (46)
	Public transportation		1 (3)
**How do you usually get to and from the following locations?**			
	**School**		
		Walk	19 (58)
		Bicycle	8 (24)
		Public transportation	0 (0)
		Drive	6 (18)
	**Social engagements (parties, religious events, concerts, sporting events)**		
		Walk	6 (18)
		Bicycle	2 (6
		Public transportation	1 (3)
		Drive	23 (70)
		Other	1 (3)
	**Work**		
		Walk	11 (33)
		Bicycle	4 (12)
		Public transportation	0 (0)
		Drive	17 (52)
		Other	1 (3)
	**Stores or shops**		
		Walk	1 (3)
		Bicycle	4 (12)
		Public transportation	0 (0)
		Drive	27 (82)
		Other	1 (3)
**What obstacles are the most challenging in using bicycles for transportation purposes (select all that apply)?**			
	Safety concerns		10 (30)
	Lack of dedicated bike paths		10 (30)
	Decreased convenience		12 (36)
	Time		13 (39)
	Physical exertion		4 (12)
	Inclement weather		14 (42)
	Cost		6 (18)
	Perceived negativity associated with biking		1 (3)
	**Other (open-ended)**		
		Lack of bike racks at destination	1 (3)
		Time of day and darkness or cold	1 (3)
**Do you consider using a bicycle for transportation to be viewed negatively among your peers?**			
	Definitely not		17 (52)
	Probably not		11 (33)
	Might or might not		4 (12)
	Probably yes		1 (3)
	Definitely yes		0 (0)
**What benefits do you see in using bicycles for transportation? (open-ended)**			
	Cheaper		15 (45)
	Environmentally friendly		12 (36)
	Exercise		23 (70)
	Fun		5 (15)
	Faster than walking		5 (15)
	Can get exercise and transport at same time		4 (12)
	No time spent looking for parking		3 (9)

### Transportation Methods

Responses to survey items about transportation methods revealed that 61% (20/33) of individuals owned a bicycle, while 73% (24/33) owned a car or truck and no participants owned an e-bike. Participants reported using a car or truck for transportation for an average of 17 days (median: 20 days) in a normal month and using a bike an average of 8.52 days (median: 1 day). Driving a standard motorized vehicle and walking were the 2 most frequent methods of transportation among study participants. Only 15% (5/33) of individuals reported biking as their most frequent method of transportation, but approximately one-third (12/33) indicated they rode a bike 2 or more times per week. A total of 7 participants indicated that they had previously ridden an e-bike. The majority of participants either walked or biked to school (university); however, most participants reported driving to social engagements, work, and stores or shops. When asked about which obstacles prevented riding a bicycle, 42% (14/33) identified inclement weather, followed by time, decreased convenience, lack of dedicated bike paths, and safety concerns. Finally, when asked about the perceived benefits associated with using a bicycle for transportation, 70% (23/33) of respondents cited exercise. Complete information on transportation methods can be found in Table 2.

### Attitudes

Participants generally felt that using conventional bicycles and e-bikes for transportation purposes would help the environment, benefit their physical health, benefit their mental or emotional health, and save them money (Table 3). Paired *t* test analysis revealed that participants believed that a conventional bicycle was more likely than an e-bike to benefit their physical health (*P*=.002) and save them money (*P*=.005). Conversely, participants believed that the e-bike was more likely than a conventional bicycle to save them time (*P*<.001).

Participants also generally felt that improving the environment, improving their physical health, improving their mental or emotional health, saving money, and saving time were “extremely good” (Table 4). Paired *t* test analysis showed that these feelings did not change from the conventional bicycle ride to e-bike ride (all *P* values >.05). Note that a few participants elected not to respond to these questions, as indicated in the table.

### Subjective Norms

When asked about the subjective norms related to riding a conventional bicycle or an e-bike for transportation purposes, participants generally agreed that their parents, friends, people who care about them, and people they look up to are supportive of them (Table 5). Paired *t* test analysis indicated that these feelings did not change when comparing the conventional bicycle with the e-bike (all *P* values >.05).

### Perceived Behavioral Control

Paired *t* test analysis revealed that compared with their views after riding the conventional bicycle, participants significantly agreed more with the statements that they could ride an e-bike on most days (*P*=.006), in the cold (*P<*.001), when they are tired (*P*=.007), when they are dressed in formal attire (*P<*.001), when carrying personal effects (backpack, groceries, books, etc; *P*=.03), on longer trips (*P*=.006), and on steep or hilly terrain (*P*<.001).

**Table 3:** Behavioral beliefs (N=33).

Behavioral belief^a^		Descriptive statistics, mean (SD)		Paired *t* test: bike versus e-bike	
		Bike	E-bike	Mean difference	*P* value
**Complete the following statement: Riding a bike or an e-bike for transportation purposes would…**					
	Help the environment	1.48 (0.67)	1.39 (0.56)	0.09	.52
	Benefit my physical health	1.09 (0.29)	1.58 (0.79)	−0.48	.002
	Benefit my mental or emotional health	1.33 (0.54)	1.42 (0.56)	−0.09	.41
	Save me money	1.42 (0.66)	1.94 (0.93)	−0.52	.005
	Save me time	3.18 (1.10)	2.12 (1.02)	1.06	<.001

^a^Variables were coded using the following logic: 1=extremely likely, 2=somewhat likely, 3=neither likely nor unlikely, 4=somewhat unlikely, 5=extremely unlikely.

**Table 4:** Outcome evaluations (N=33).

Outcome evaluation^a^		Descriptive statistics, mean (SD)		Paired *t* test: bike versus e-bike	
		Bike	E-bike	Mean difference	*P* value
**Please note your feelings toward the following statements:**					
	Improving the environment is…	1.27 (0.52)	1.23 (0.43)^b^	0^b^	>.99^b^
	Improving my physical health is…^c^	1.03 (0.18)^c^	1.03 (0.18)^b^	0^d^	>.99^d^
	Improving my mental or emotional health is…	1.00 (0)	1.07 (0.25)^d^	−0.07^d^	.16^d^
	Saving money is…	1.09 (0.29)	1.09 (0.30)^c^	0^c^	>.99^c^
	Saving time is…	1.21 (0.48)	1.19 (0.40)^c^	0.03^c^	.57^c^

^a^Variables were coded using the following logic: 1=extremely good, 2=somewhat good, 3=neither good nor bad, 4=somewhat bad, 5=extremely bad.

^b^n=31.

^c^n=32.

^d^n=30.

**Table 5:** Subjective norms (N=33).

Subjective norms^a^		Descriptive statistics, mean (SD)		Paired *t* test: bike versus e-bike	
		Bike	E-bike	Mean difference	*P* value
**Please note your feelings toward the following statements:**					
	My parents are supportive of me riding a bike or an e-bike for transportation purposes	1.06 (0.24)	1.12 (0.33)	−0.06	.16
	My friends are supportive of me riding a bike or an e-bike for transportation purposes	1.21 (0.42)	1.12 (0.33)	0.09	.08
	People who care about me are supportive of me riding a bike or an e-bike for transportation purposes	1.09 (0.29)	1.09 (0.29)	0	>.99
	People I look up to are supportive of me riding a bike or an e-bike for transportation purposes	1.09 (0.29)	1.18 (0.39)	−0.09	.08

^a^Variables were coded using the following logic: 1=true, 2=false.

**Table 6:** Perceived behavioral control (N=33).

Perceived behavioral control^a^		Descriptive statistics, mean (SD)				Paired *t* test: bike versus e-bike			
		Bike		E-bike		Mean difference		*P* value	
**I believe that I can ride a bike or an e-bike for transportation purposes...**									
	Most days		1.97 (0.88)		1.55 (0.71)		0.42		.006
	In the cold		3.15 (1.25)		2.52 (1.25)		0.64		<.001
	In the heat		1.94 (0.93)		1.73 (0.94)		0.21		.15
	In the rain		3.3 (1.38)		3.09 (1.26)		0.21		.27
	In the snow		4.15 (1.12)		4 (1.15)		0.15		.30
	In the daylight		1.21 (0.48)		1.09 (0.29)		0.12		.10
	In the dark or at night		2.52 (1.33)		2.15 (1.23)		0.36		.08
	When I am tired		2.39 (1.12)		1.88 (0.93)		0.52		.007
	When I am dressed in casual attire		1.3 (0.59)		1.21 (0.42)		0.09		.37
	When I am dressed in formal attire		3.45 (1.3)		2.7 (1.26)		0.76		<.001
	When traffic is heavy		2.42 (1.17)		2.18 (1.18)		0.24		.19
	When traffic is light		1.42 (0.9)		1.24 (0.61)		0.18		.08
	When there is a dedicated bike lane		1.27 (0.45)		1.18 (0.39)		0.09		.26
	When there is a dedicated bike path		1.15 (0.36)		1.09 (0.29)		0.06		.32
	When I am rushed or in a hurry		2.85 (1.39)		2.33 (1.34)		0.52		.05
	When I am with a group of friends		3.24 (1.37)		2.82 (1.47)		0.42		.06
	When carrying personal effects (backpack, groceries, books, etc)		2.97 (1.38)		2.48 (1.28)		0.48		.03
	On shorter trips (<1 mile)		1.36 (0.90)		1.36 (0.70)		0		>.99
	On longer trips (>1 mile)		2.12 (1.11)		1.61 (1.06)		0.52		.006
	On flat terrain		1.09 (0.29)		1.12 (0.33)		−0.03		.57
	On steep or hilly terrain		2.42 (1.15)		1.64 (0.93)		0.79		<.001

^a^Variables were coded using the following logic: 1=strongly agree, 2=somewhat agree, 3=neither agree nor disagree, 4=somewhat disagree, 5=strongly disagree.

### Distance, Time, Speed, and Heart Rate Metrics

**Figure 1:**
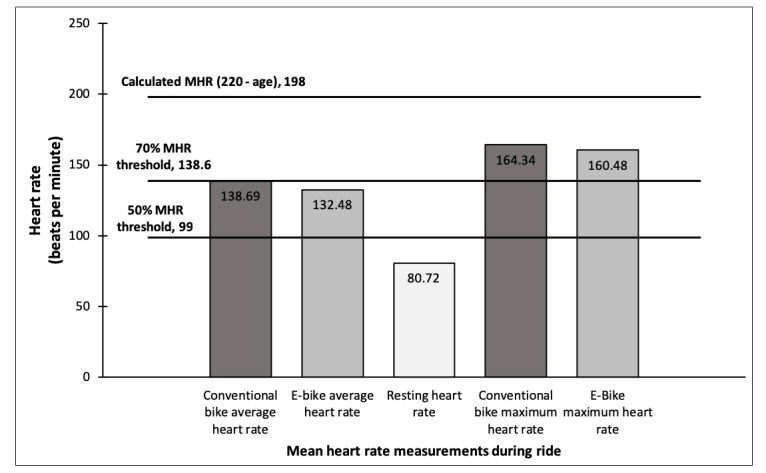
Comparison of heart rate metrics. MHR: maximum heart rate.

Participants traveled approximately 10 miles (approximately 16 km) while following the dedicated path. Paired *t* test analysis (Table 7) revealed participants completed the course on an average of 14 min and 34 s faster when using e-bikes as opposed to conventional bicycles (*P*<.001). The mean average speed of travel on the e-bike was faster than on the conventional bicycle (*P*<.001), as was the mean maximum speed of travel on the e-bike (*P*<.001). Participants’ mean average heart rate during the e-bike ride was lower than that during the conventional bike ride (*P*=.04; see Figure 1). The average heart rate above resting during the e-bike ride was 51.76 beats per minute (bpm), which is 89% (51.76/57.97) of the average heart rate above resting during the conventional bike ride. When looking at the mean heart rate by gender, the trends were similar in direction and magnitude. In paired *t* test analyses, the mean average heart rate for males and females during the e-bike ride was lower than that during the conventional bike ride; however, these findings were not statistically significant (*P*=.11 and *P*=.24, respectively). The mean MHR of participants during the rides was higher while on the conventional bicycle compared with the e-bike, but this difference was not statistically significant (*P*=.26). The mean average heart rate during both the conventional bike ride and the e-bike ride was faster than the mean resting heart rate. Both were also significantly higher than the resting heart rate (*P*<.001). With a mean age of 22 years, participants’ estimated MHR was 198 bpm (this was calculated using the formula 220−age as described in the Methods). The target average heart rate range for moderate-intensity exercise (50%-70% of MHR) was then calculated to be 99 bpm to 138.6 bpm (0.5×198=99; 0.7×198=138.6) [38].

**Table 7:** Comparison of distance, time, speed, and heart rate metrics (n=31).

Metric	Descriptive statistics, mean (SD)		Paired *t* test: bike versus e-bike	
	Bike	E-bike	Mean difference	*P* value
Ride duration (minutes:seconds)	53:37 (10:55)	39:02 (6:24)	14:34	<.001
Average speed (miles per hour)	12.26 (1.94)	16.37 (2.27)	−4.11	<.001
Top speed (miles per hour)	21.86 (3.96)	27.02 (2.44)	−5.15	<.001
Average heart rate^a^ (bpm^b^)	138.69 (16.59)	132.48 (14.10)	6.21	.04
Maximum heart rate^a^ (bpm)	164.34 (17.74)	160.48 (16.31)	3.86	.26
Resting heart rate^a^ (bpm)	80.72 (15.02)	N/A^c^	N/A	N/A
Resting heart rate versus average heart rate (conventional bike ride)^a^ (bpm)	N/A	N/A	−57.97	<.001
Resting heart rate versus average heart rate (e-bike ride)^a^ (bpm)	N/A	N/A	−51.76	<.001

^a^n=29.

^b^bpm: beats per minute

^c^N/A: not applicable.

## Discussion

### Principal Findings

The purpose of this study was to compare e-bikes with conventional bicycles in answering the following questions: (1) what proportion of the health benefits are retained when using an e-bike as compared with using a conventional bicycle? and (2) what are the attitudes and beliefs toward e-bikes after riding one and how do those compare with attitudes and beliefs toward conventional bicycles? While significant differences in heart rate were measured between conventional bicycle and e-bike use, results indicate that both equated to significantly higher heart rates than were recorded at rest. In particular, when using average heart rate as a proxy for the health benefits of cycling, e-bike use retained 89% of the cardiovascular health benefits gained from riding a conventional bike. Furthermore, mean scores indicate that participants’ average heart rate was well within the target heart rate range of 50% to 70% of MHR for moderate-intensity physical activity (132.48 bpm, or approximately 67% [132.48/198] of estimated MHR) while riding the e-bike [38]. Therefore, e-bike use in this study retained the majority of the cycling cardiovascular health benefits and met established biometric thresholds for cardiovascular fitness. This finding is comparable with a similar finding in another study, in which e-bike users reached a mean heart rate of 69% and 67% of an estimated MHR in the ECO (eco support) and POW (power support) of the e-bike models used [35]. The findings from this study confirm the findings in previous studies that e-bikes can satisfy requirements for moderate-intensity physical activity [10,12,13,31,34-36]. In particular, the trends of the mean heart rate measurements were comparable with other studies, with the e-bike mean heart rate being lower than the mean heart rate on a traditional bike or a bike without electric motor assistance [34,35]. However, mean heart rate measurements did vary somewhat, which may be explained by the participants being required to stop and get off the bike in one study (lower mean heart rates) [35] or the hilly environment of the other study (higher mean heart rates) [34].

In general, participants’ attitudes toward conventional bicycles differed in several distinct ways as compared with e-bikes. In relation to physical health benefits and cost-saving measures, participants favored the conventional bicycle. These findings are understandable as heart rate results did indeed show that riding the conventional bicycle required increased physical exertion, and the retail price of the e-bikes used in this study was approximately 5 times higher than the retail price of the conventional bicycles. However, participants reported comparatively more favorable attitudes toward e-bike use on several survey items. First, participants indicated that e-bikes were more likely to save them time—a belief backed by the results showing an increase in speed when riding an e-bike. Next, participants indicated they were more likely to ride e-bikes for everyday use in adverse conditions, including cold weather, when physically tired, when dressed in formal attire, when carrying personal effects, on longer trips, and on steep or hilly terrain. When taken together, these results demonstrate a belief that e-bikes are easier to ride, similar to the finding in a previous study that e-bikes are more enjoyable to ride and result in lower levels of perceived exertion [31]. Therefore, this study supports the idea that e-bikes may act as a catalyst in helping individuals clear some of the personal barriers to active transport cycling. Additional research in this area may be useful to understand the causes of these attitudes, including separately analyzing data from individuals who own bicycles, prefer bicycles as a mode of transportation, and those who use them frequently.

### Limitations

Challenges encountered during the bicycle-riding portion of this study included technical difficulties with the e-bikes and the Apple Watches. During the e-bike rides, 2 participants experienced technical difficulties with the e-bikes in which the batteries were not functioning properly and, therefore, not providing assistance for the entire duration of the ride. Because of this, the time, speed, distance, and heart rate measurements for these 2 participants were excluded from the analysis. These issues may have also affected the participants’ views of the bikes’ functionality. In addition, the heart rate measurement function of the Apple Watches did not work properly for 2 participants’ and their heart rate measurements were, therefore, excluded from the analysis. Also, GPS tracking data gathered through the Strava app on each Apple Watch are prone to some error, yielding distance measures that varied slightly between participants, despite all participants riding the same route. These variations were examined and determined to be random, equally distributed across both the conventional and e-bike rides, and impacted measures by less than 0.2 miles over the course of the ride.

One potential bias in this study is a social desirability bias. Social desirability bias drives an individual to answer in a way that makes them look more favorable to the experimenter or to society. Participants may have sensed that researchers wanted the e-bikes to be viewed more positively and may have answered accordingly. The surveys were, however, administered online so the participants could answer questions in private. A future study could randomly assign some participants to ride the e-bike first, and some to ride the normal bike first, to mix up the order and perhaps reduce this bias. Recall bias may also have influenced participants’ responses, as participants took the surveys at varying times and may have remembered their experience differently as a result of this variation. This, however, is not expected to be a large bias, as participants are likely to accurately remember such a unique experience.

The survey that collected demographic information asked participants to report a combined annual household income. Despite being university students, 6 participants reported household incomes above US $100,000, likely because they were still living at home and reported their parents’ incomes. A future study among university students should ask for a personal income, as that will likely be of greater interest to researchers. Finally, the study population was neither very diverse nor very large, even for a pilot study, making its findings less generalizable to other populations. A future, larger study should seek out a more diverse population regarding age, race or ethnicity, and income level. The limited sample for this pilot study is not likely to have impacted the biometric estimates, but it could have had an effect on the measures of attitudes and beliefs.

### Conclusions

This pilot study suggests that e-bikes are an active form of transportation capable of providing much of the cardiovascular health benefits obtained during conventional bike use. Participants reported that they were more likely to use an e-bike for everyday transportation than a traditional bike and were still able to meet established criteria for moderate-intensity physical activity during e-bike use. While still providing an opportunity for physical activity, these findings suggest that e-bikes may help reduce several key personal factors known to be obstacles to conventional bike use, such as increased transportation time, decreased convenience, and physical fatigue. These findings also suggest that public health officials should advocate for the daily use of e-bikes as a novel means of meeting physical activity recommendations through active transportation, all while mitigating the effects of traditional barriers to active transport cycling. E-bike manufacturers could also frame their product development and marketing practices in light of these findings by seeking to develop more cost-friendly e-bike options and by marketing their e-bikes as a means of reaching activity guidelines while avoiding inconveniences of traditional cycling. E-bike manufacturers could also expand their marketing to individuals who may be otherwise reluctant to engage in physical activity, such as older or overweight individuals.

As this is a pilot study, these results would benefit from being confirmed in a larger and more representative sample. In addition, future studies would benefit from including other energy-expenditure outcome measures, such as human power output and tests related to oxygen consumption. Future research should explore how e-bike use might improve environmental health indicators by potentially decreasing reliance on standard motorized vehicles and fossil fuels, decreasing noise and air pollution, and relieving traffic and parking congestion. Future studies would benefit from the application of a similar research design to populations who may be less inclined to use active forms of transportation, such as older individuals, obese or overweight individuals, and those who may be impacted by physical injury or impairment. In addition, this study could also be extended to the use of electric pedal-assist mountain bikes, or eMTBs, on soft-surface and off-road trails.

## Abbreviations

bpm: beats per minute
E-bike: electric pedal-assist bicycle
eMTB: electric pedal-assist mountain bicycle
GED: General Educational Development
GPS: global positioning system
MHR: maximum heart rate
TPB: theory of planned behavior
